# Discovering Genetics‐related Biomarkers of Neuronal Vulnerability Using Post‐mortem Datasets from Multiple Large Brain Banks

**DOI:** 10.1002/alz70856_098053

**Published:** 2025-12-24

**Authors:** Xiaolin Zhou

**Affiliations:** ^1^ University of Toronto, Toronto, ON, Canada

## Abstract

**Background:**

Neuropsychiatric disorders are linked to changes in brain composition, such as cortical neuron loss in late‐onset Alzheimer's disease (AD). GABAergic cell types have been implicated in dysregulated neural interactions, contributing to pronounced neurodegeneration. However, the inaccessibility of human brain biopsies hampers understanding of cell type proportion changes in neuropsychiatric disorders. This study aims to identify genetic factors governing cell‐type vulnerabilities and their links to neuropsychiatric traits. We hypothesize that the selective vulnerability of neuronal subtypes is associated with distinct genetic risk factors.

**Method:**

Cell‐type deconvolution algorithms for bulk RNAseq data from the Religious Orders Study/Memory and Aging Project (ROSMAP) study (*n* = 912) were benchmarked and validated against single‐nucleus RNA sequencing (snRNA‐seq) data from the same subjects. A genome‐wide association study (GWAS) was conducted to identify genetic variants linked to changes in cell type proportions (CTPs).

**Result:**

We found that no single deconvolution method is universally effective across all cell types and datasets. The MarkerGeneProfile (MGP) method demonstrated relatively high accuracy in predicting cell type proportions. Incorporating approaches to account for technical covariates in RNAseq data significantly improved the power of GWAS analyses, identifying genetic variants associated with changes in CTPs.

**Conclusion:**

This research demonstrates that brain cell type proportions can be reliably inferred from bulk brain tissue RNAseq datasets. Furthermore, our findings reveal that these proportions are partially governed by genetic factors, advancing our understanding of neuronal vulnerability in neuropsychiatric disorders.

